# A Text-Book of Embryology for Students of Medicine

**Published:** 1900-10

**Authors:** 


A TEXT-BOOK OF EMBRYOLOGY FOR STUDENTS OF MEDICINE.—By John Clement Heisler, M. D., Professor of Anatomy in the Medico-Chirurgical College, Philadelphia. With 190 illustrations, 26 of them in colors. 405 pages. Price, $2.50. Philadelphia: W. B. Saunders, 925 Walnut Street. 1899.

This book is intended to give to the student of medicine a clear and comprehensive view of embryology. The text is elaborate enough to meet the demands of schools, and the arrangement of the subject matter is simple and convenient. There is no work on the market more valuable to the student of medicine than this one.

